# Reinforcement Emotion-Cognition System: A Teaching Words Task

**DOI:** 10.1155/2019/8904389

**Published:** 2019-05-02

**Authors:** Minjia Li, Lun Xie, Anqi Zhang, Fuji Ren

**Affiliations:** ^1^School of Computer and Communication Engineering, University of Science and Technology Beijing, Beijing 100083, China; ^2^Hefei University of Technology, Hefei, China

## Abstract

The goal of this paper is to suggest a system for intelligent learning environments with robots modeling of emotion regulation and cognition based on quantitative motivation. A detailed interactive situation for teaching words is proposed. In this study, we introduce one bottom-up collaboration method for emotion-cognition interplay and behaviour decision-making. Integration with gross emotion regulation theory lets the proposed system adapt to natural interactions between students and the robot in emotional interaction. Four key ideas are advocated, and they jointly set up a reinforcement emotion-cognition system (RECS). First, the quantitative motivation is grounded on external interactive sensory detection, which is affected by memory and preference. Second, the emotion generation triggered by an initial motivation such as external stimulus is also influenced by the state in the previous time. Third, the competitive and cooperative relationship between emotion and motivation intervenes to make the decision of emotional expression and teaching actions. Finally, cognitive reappraisal, the emotion regulation strategy, is introduced for the establishment of emotion transition combined with personalized cognition. We display that this RECS increases the robot emotional interactive performance and makes corresponding teaching decision through behavioural and statistical analysis.

## 1. Introduction

One of the most significant discussions of intelligent interactive robotics is the collaboration of cognition and emotion adjustment. The internal processes of emotion-cognition interplay appear in the form of behaviour performance, and positive objects (with a high valence value) tend to be more acceptable than negative ones [1]. Agents have more effective interactions with the human in emotional scenes than nonintelligent machines, which leads to raising of users' enthusiasm for operation.

Relationship between emotion and cognition is bidirectional [2]. Emotion influence on cognition has three major compositions: perception [3], attention [4], and memory [5]. However, some researches evidence that the activation of emotion is acted on by cognitive process [6]. Lewis [7] establishes a framework to describe this relationship through the lens of dynamical systems theory. In previous works, the emotion-cognition collaboration has been implemented through the development of competing and complementary computational models [8]. Typical models, such as OCC (Ortony, Clore, and Collins) [9] and Scheutz and Sloman [10], direct the emotion-cognition collaboration towards resources allocation problem to address the control choices. In our work, we emphasize the collaboration effect on the goal to enhance positive emotional interaction experience through integration with emotion regulation and provide a detailed teaching English words task to validate the effectiveness of the system.

This study is conducted to provide an intelligent learning environment for students and robotic teachers with the emotion-cognition collaboration ability. The robotic teacher can be enabled to select an emotional facial expression and provide a difficulty level of the next English words' meaning multiple choice question. The difficulty level is more in line with robot's preference according to the student's current performance. The robotic teacher focuses on two external factors: the students' scores and their emotional state. Moreover, it has two intrinsic factors: motivation and emotion, which could be a cooperative and competitive relationship, jointly determining the behaviour output.

Our paper is organized as follows. In Section 2, we explain the relationship between cognition, motivation, and emotion and introduce some related work about emotional modeling and regulation.  Section 3 provides a whole system structure.  Section 4 explicitly describes the experiment and methodology of teaching words task.  Section 5 presents the experimental results of the proposed method. Finally, the suggestions for future development are mentioned in Section 6, and a conclusion is presented in Section 7.

## 2. Related Works

### 2.1. Cognition, Motivation, and Emotion

In cognitive engineering and cognitive psychology, cognition is generally assumed to be a participant in information processing [11]. Motivation can be treated as a desire to perform an action while emphasizing on behaviour output and adjustment. And, in some researches [12], motivated cognition is provided for thinking in ways that produce conclusions consistent with one's desires, which blurs the information processing boundaries of cognition and motivation.

Like cognition and emotion, emotions and motivation are related structures but are not exactly the same [13]. Studies by Linnenbrink and Pintrich [14] indicate that emotions can not only be based on cognitive processes but can also have a powerful effect on these motivational processes. While some people think that emotions and motivations are indivisible [15, 16], many believe these constructs are related but differentiated. For example, the Psi (principle of synthetic intelligence) theory considers that emotions are not separate from motives, memories, etc. and are not independent modules in the cognitive system [17]. In detail, changes in emotional state occur and alter their subsequent motivation, hindering or facilitating the achievement of the goal [18]. Cognitive processing is also an integral part of emotions and motivations that affect how often they affect ongoing activities and behaviours. It is becoming more and more clear that there is a complex intertwined cognition, emotion, and motivation, which makes it difficult to determine the boundaries between them [19].

Motivation has neurobiological origins in basal ganglia and midbrain marginal dopaminergic pathways. Chumkamon et al. [20] made synthetic dopamine from a sample of rat dopamine for motivational stimulation of long-term memory. It indicates that first occurrence of an incident will cause high motivation (or be seen as “novelty”). However, when the same accumulated stimulation occurs repeatedly, it tends to be less valuable (“boring”) [21]. Quantitative analysis for understanding motivation in this paper provides a method to measure external stimulus extracted from sensorimotor, driven by the goal priority, action selection, reward, or feedback. And, the results from these processes are just initial motivation without emotion modulation.

Moreover, emotion is one kind of internal state constantly motivated and experienced by the individual. For the reason that emotion is awoken consciously or unconsciously and can be considered an emergent property of motivationally driven neural activity [13], the emotion-cognition collaboration considers receiving initial motivation to generate a current emotional state. Thus, the emotional response of individual coordinates with environments changes. The influence of emotion on motivation focuses on a higher fluctuation of emotion causing more emotional output propensity, measured by internal reward signals in reinforcement learning. This paper suggests an accumulated value of first derivative as sifting the moment that emotion wins the competition.

### 2.2. Emotional Modeling

In the recent literature, emotional modeling is motivated by two main theories: anatomical approach [22] and appraisal theory [23]. The former focuses on the establishment of emotional brain-inspired neural networks and is beneficial to nonlinear or uncertainty prediction of engineering. Emotional neural networks (ENNs) are normally composed of four modules: amygdala, orbitofrontal, sensory cortex, and thalamus, and have a conditional learning process related to external emotional stimuli [24]. The most representative achievement is the brain emotional learning (BEL), which has been successfully utilized in pattern recognition and complex control application. For example, the term “brain emotional learning-based intelligent controller” (BELBIC) proposed by the Lucas et al. [25] has been applied for some SISO, MIMO, and nonlinear systems. Lotfi and Akbarzadeh-T. [26] proposed the brain emotional learning-based pattern recognizer (BELPR) for chaotic time-series prediction problems. In addition, there are some studies improving ENN structures. Lotfi et al. [27] established a winner-take-all rule in the sensory cortex, feeding orbitofrontal, and amygdala to solve nonlinear problems in the design of a tensegrity structure. The competitive BEL (C-BEL) [28] method was proposed for solving *n*-bit (≥3) parity problems, inspired by the neurocircuits' competitive property.

Appraisal theory emphasizes on the dynamic emotional processes, considering that emotion derives from a person's cognitive interpretation of environmental relations [29]. These typical computational models based on appraisal, e.g., OCC [9], EMA [29], FLAME [30], and ALMA [31], treat cognition as an indispensable foundation for emotional computing models and are used in the process of event-emotion mapping. Appraisal theory aims to simulate the dynamic changes of emotions when events occur. Emotions are thought to be produced by individual judgment patterns. This is an exploration of the relationship between people and the environment. Unlike appraisal theory, anatomical approach tends to foreground certain process assumptions. Therefore, the RECS framework is established by appraisal theory instead of anatomical approach. In recent years, there have been many emotional computing models and cognition-emotion models based on appraisal theory. The emotion elicitation conditions (EECs) model [32] used fuzzy logic to predict the emotional state based upon event appraisal. Rodríguez et al. [33] proposed a software systems, based on the purpose of extensive interaction, to generate the emotionally driven responses considering about cognitive component. But cognition is treated as an intermediate process between emotion and behaviour, and it lacks the ability to describe more complex cognition-emotion collaboration. We consider about the competition between motivation and emotion to achieve the automatic transformation of goal-directed behaviour in a specific teaching environment.

### 2.3. Emotion Regulation

In the natural emotional interaction, the emotional change will be affected by a series of external and internal factors. It is affected not only by external emotional stimulation and the current emotional state of the impact but also by the individual's own emotional cognitive ability.

Appraisal theorists typically treat appraisal process as the cause of the emotion, or at least of the physiological, behavioural, and cognitive changes associated with emotion [23, 34, 35]. Gross emotion regulation theory [36] based on the individual's cognitive ability to understand the event changes the emotional experience so as to rationalize this matter, which is the key to the emotional regulation process. Gross argues that the process of emotional regulation consists of five parts: situation selection, situation modulation, attention distribution, cognitive reappraisal, and response inhibition. Among them, the first two are based on changes from the external environment; the rest is for the individual subjective will or behaviour carried out.

Cognitive reappraisal occurs before the emotional response, and the emotional state is reappraised and adjusted; expression inhibition occurs after the emotional response behaviour. Cognitive reappraisal strategy as a priority regulation strategy reduces the negative emotional experience better, and emotional state tends to be alleviated. Gross suggests that emotion regulation refers to the process of influencing emotions, experiences, and expressions. More generally, emotion regulation involves the change of emotional latency, time, duration, behaviour expression, psychological experience, physiological response, etc. Therefore, we establish a collaboration system to describe the dynamic process. Thompson [37] argues that “emotional regulation refers to the intrinsic and extrinsic process of monitoring, assessing, and correcting emotional responses that individuals make to accomplish their goals.”

For intelligent learning environment, advanced robotic teachers need emotion regulation ability, which helps robots generate more positive emotional state, while ensuring the smooth transition of emotions and modest changes based on cognition.

### 2.4. Reinforcement Emotion-Cognition System

**Figure 1:**
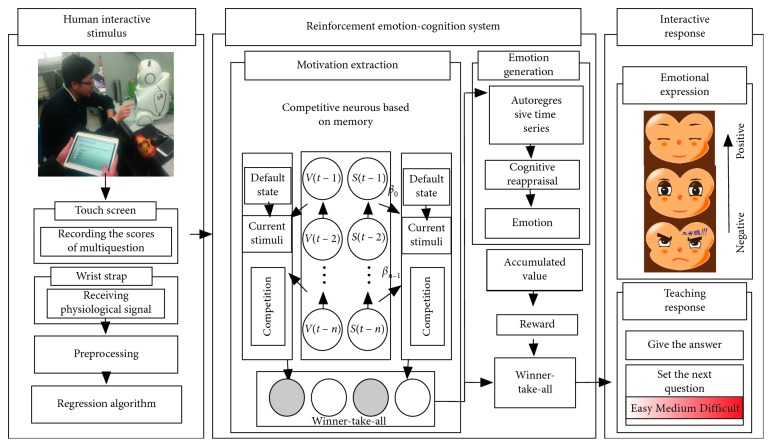
Overview of the proposed methodology: the RECS structure.

Our research rather lies in a system level.  Figure 1 shows the whole RECS structure. Multilevel emotional response and emotion regulation are based on cognition. The information processing flow involves parallel computational processes allowed for shifting to the action space. It contains memory storage (update competitive neurons), cognitive allocation (motivation extraction), and conditioning (emotion regulation and behaviour decision), which refers to prediction and classification. Various behaviours are triggered on the basis of different levels of stimulus. In this paper, the bottom-up stimulus extraction module obtains interacting user's emotional label valence_H_ by support vector regression (SVR) through detected physiological signal, and the information on the user's operations is obtained through the touch screen; cognitive structure and emotion generation generate responses emphasized on emotion regulation; behaviour decision relies on the competition (Winner-Take-All) results of emotion or initial motivation.

## 3. Teaching Words Task

### 3.1. Method

Frenzel [38] describes the relationship between teacher emotions and student behaviour responses. Their model suggests that teacher's emotions are influenced by student behaviour, which in turn affects teaching. Besides, many researchers prove that teachers' emotions have different effects upon students [39]. “we could affirm that positive emotions have positive effects and negative emotions have negative effects on students.” Based on this, we present a scenario for interaction between an autonomous RECS robot and one EFL (English as a foreign language) learner, designed to consider the robot's emotion-cognition collaboration in teaching word tasks, laying the foundation for further supporting the development of the learner's self-efficacy in the process of learning words. The representation of the motivation of the collected learner's feedback can be used to control robots' emotion and goal-driven teaching contents and their level of difficulty, while the robotic emotion triggered by initial motivation through emotion regulation can in turn affect the students' emotion. The robot has two drives: learners' physiological states refer to physical pressure and emotion and its own expectation to achieve more teaching tasks. Of particular interest in terms of behavior selection will be the situations where the selection of questions' difficulty in teaching and emotional facial expression is according to the robot's inner real emotion.

**Figure 2:**
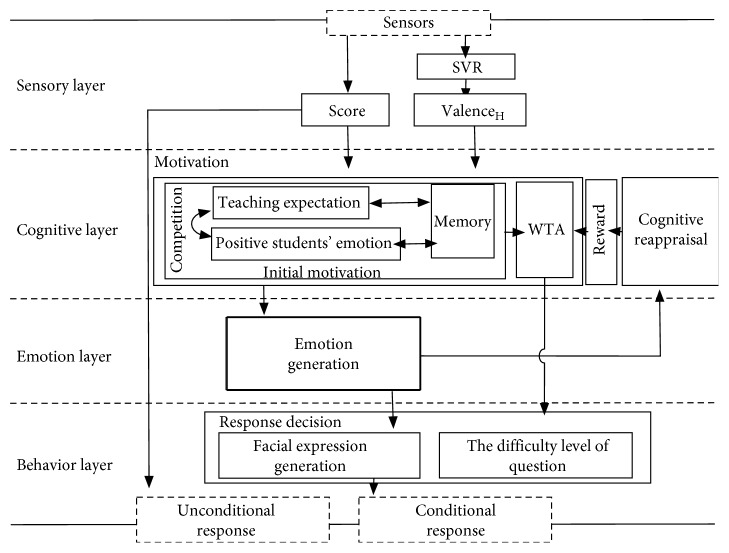
The detailed bottom-up hypothetical layers of emotion-cognition collaboration.

Our collaboration system tends to research the effect of emotion regulation on interaction.  Figure 2 gives a more detailed illustration of the hypothetical layers that implement this model, with an emphasis on motivation extraction, emotional regulation, and response decision (including behaviours and facial emotional expression). The inputs from sensors contain learner's physiological signals and the choices for the multiple-choice question of word meaning. The stimulus signals are given by recognized valence (valence_H_), which refers to the emotional pressure of learners and whether the answer is correct. The external factors in the cognitive layer involved are the expectation to achieve more teaching tasks and learner's state composed of emotional pressure. There is competition between the two goals, based on the output expectation vs. learner's pressure, to determine the next phase of the study. The reinforcement learning process is used to measure the emotional influence on the final motivation. In other words, the learner's emotion factor also helps to achieve the expectation. It appears in the robot's internal emotion regulation that learner's negative emotion leads to delay in teaching process. And, robotic positive emotional state generated by emotion regulation can be detected by RL. Last, in the behaviour layer, the unconditional response shows the words meaning answer, and for the conditional response, the robot has two kinds of behaviour: selection of words according to the difficulty level and the facial expression representing the real emotional state. Generally, the emotion regulation occurs on an emotion-cognition collaboration level but appears in the expression level.

### 3.2. Experimental Setup

In order to validate the research instrument, we invited 24 Chinese undergraduates of the same 20 years of age. Before taking part in the study, all the undergraduates had received more than six years of formal EFL education and passed the EFL test of the National College Entrance Examination in China with same scores.

We used a MATLAB simulator to achieve perception and reinforcement learning process. This experiment was performed on a noninvasive physiological collected wrist strap and a robotic platform with one contact interface, one loudspeaker, etc. For valence extraction, we used DEAP dataset for the training model. Besides, the communication protocol between the robot and wrist strap is Bluetooth 4.0.

The specific experiment steps are as follows:

1. Each student makes sure these subjects keep calm at first and then let them scan the words list without meaning, scoring according to the degree of familiarity quickly
2. According to the specific student, the words list is imported into a corresponding robot system
3. The robot teaches the word to each student by providing multiple choice questions for choosing the right meaning of the word
4. In the teaching process, the robot provides a random word at the beginning, and when it receives the feedback from the learner, it selects the difficulty level of the word and makes emotional expression based on RECS
5. Each student is allowed to answer 40 questions

As for experimental comparison, we divided the 24 students into four groups with four different configurations (the details are shown in Results) with six students per groups. Each group has different system parameters leading to discrepant emotional state and behaviour output.

## 4. Methodology

### 4.1. Bottom-Up Stimulus Extraction

The stimulus in the interaction process derives not only from sensory level but also from analysis of motivation. For teaching words task, the stimulus is obtained from two modules: interactive stimulus (scores) and valence extraction. The emotion of interactive students could influence agent's cognition. Thus, we extract valence characterized as the emotion which is measured as the positive degree. More specifically, emotionally valenced (e.g., pleasant-unpleasant or desirable-undesirable) sensory and physiological signals give the agents a subjective and motivated perception of their interactive behaviours. Their sensations, as well as their actions, are no longer neutral and objective but are rather emotionally coloured. In our experiment, the subjects wear supplementary noninvasive wrist straps which record physiological signal synchronously. SVR algorithm is used to recognize valence_H_ through the physiological features and train the regression model with the real-time features. The training labels (contain instantaneous labels) are self-reported even in short-time events (5 s) that ensure the accuracy of emotion prediction in real-time interaction. Apart from this, we consider that recognizing an interactive stimulus implies the recognition of the sense of students' interactive effect (a set of cognitive definition corresponding to the literal meaning and perceptive meaning). The memory unit is required to hold this information of mapping relationships and stimulus's level. The combined information is a vector recording questions' difficulty and accuracy of answers. Specific scores are artificially defined for different kinds of questions' difficulty. Once sensors receive the new external stimulus, vector's value are updated and delivered to the next layer. There are two conditions. They are as follows:

Transferring to the behaviour layer directly, i.e., the direct stimulus without cognitive and emotional modulation. In the specific task, it means giving students the right answers.

Transferring to cognitive layer and then obtaining the next question and agent's emotional expression based on emotion-cognition collaboration.

### 4.2. Cognitive Layer

As the previous sections described, the emotion modulation happens in the cognitive layer. Thus, the cognitive structure deals not only with output as an effect of cognition to emotion and behaviour but also with input as an effect of emotion and the environmental stimulus to cognition. Agents need motivation to reduce the difference between environmental stimuli and ideals. Thereby, it can notice when the current situation is different from what it recognized. The motivation is extracted from two quantitative goals [40]: teaching expectation and positive students' emotion.

**Figure 3:**
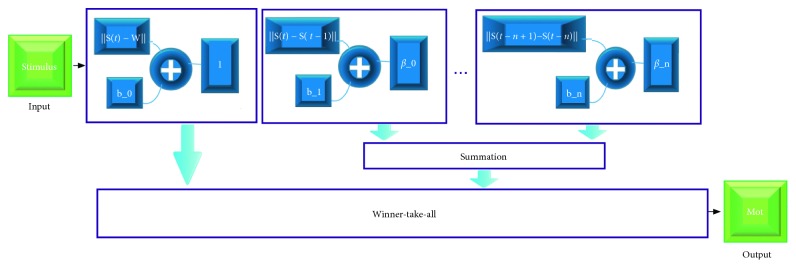
The network structure for motivation acquisition.

In neurobiology, dopamine is one of the factors that determine motivation, and there is selective depletion process of forebrain dopamine. Therefore, different from self-organized cortical cognitive maps, the presented method of motivation acquisition focuses on the evolution of the whole in the time dimension, and external stimulus still serves as the only input to the network. As shown in Figure 3, there is no connection between network layers, and each layer output points to the Winner-Take-All module.

For simplification, normalizing both ranged from (0, 1) before further calculation. In order to represent the current motivation, we rely on competitive network structure and establish competitive neurons considering about short history effect. The network is composed of two aspects: competition between current external stimulus and default inner state and competition between current differences and history differences. Thus, current difference *D*_0_ is an output of the neuron with the weights **W** and external stimulus **S**(*t*) input:

(1) $$\begin{array}{c} D_{0}=\left\|W-S\left(t\right)\right\|+{\text{bias}}_{0}, \end{array}$$

where **W** also represents inner default state of agents. We measure the effect of *t* − *i* time as follows:

(2) $$\begin{array}{c} D_{i}=\left\|S\left(t-1-i\right)-S\left(t-i\right)\right\|+{\text{bias}}_{i}. \end{array}$$

The idea of measuring novelty of prediction errors for the purpose of self-improvement has been considerably exploited in the research on intrinsic motivation [41]. But long-time similar stimulus causes lower prediction errors and motivation attenuation [42]. Thus, the motivation output is obtained by

(3) $$\begin{array}{c} \text{Mot}=\max\left(D_{0},\overset{n}{\underset{i=1}{\sum }}\beta_{i-1}\left(\left\|S\left(t-1-i\right)-S\left(t-i\right)\right\|+{\text{bias}}_{i}\right)\right), \end{array}$$

where *β* is the attenuation weight and is given as follows:

(4) $$\begin{array}{c} \beta_{i}=\exp\left(-\delta \cdot i^{2}\right), \end{array}$$

where *δ* represents the descending speed of the history effect.

**Figure 4:**
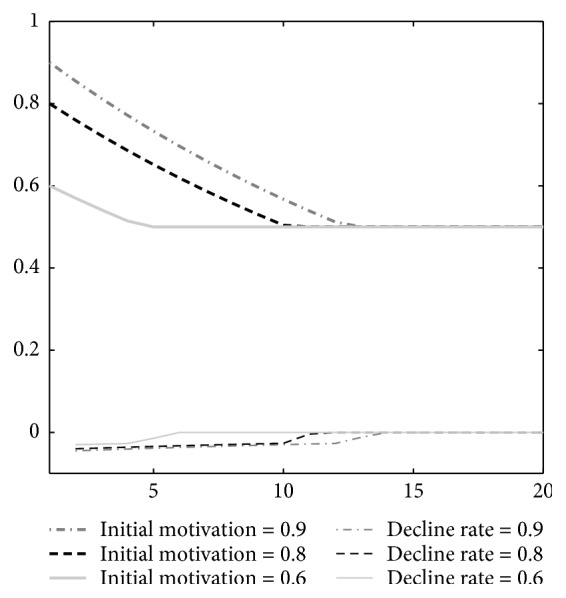
Motivation attenuation during the long-time identical external stimulus. The lines from top to bottom represent external stimulus intensity: 0.9, 0.8, and 0.6.

Figure 4 shows the motivation attenuation during the long-time identical external stimulus. The higher stimulus causes the higher initial motivation at the beginning and the slower descent speed.

### 4.3. Emotional Layer

**Figure 5:**
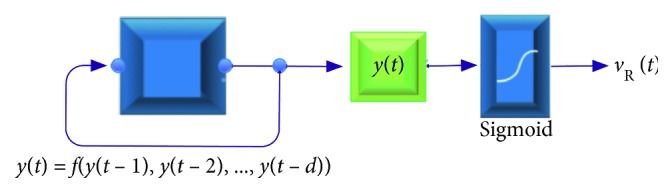
The calculation process in the emotional layer.

The sensory cortex receives a signal that is transmitted to the amygdala via the thalamus, producing an emotional state. It exists in the brain in the activity form of “feeling stream.” The inner limbic structure and the thalamic system, including specific hormones and chemical neurotransmitter activities, ensure the persistence of the feeling stream [43]. Therefore, we consider the robotic emotional transition is flat within a threshold range, which manifests itself in the tradeoff between the Euclidean distance of emotional state in emotional dimension in contiguous time and the intensity of motivation. It ensures a reasonable trend of emotional transition. The extended amygdala can transmit motivationally relevant signal to emotionally relevant hypothalamic and brainstem structures [44]. Thus, the process of emotional transfer is related not only to the emotional state of the previous moment but also to the current motivation. The emotional trajectories can be treated as autoregressive time-series process.  Figure 5 shows the emotion generation process using the autoregressive model. *y*(*t*) is the intermediate value participating in the time-series process, and sigmoid function is used to ensure that the emotional output sequence is between 0 and 1.

This module can be written as a simple Taylor expansion to represent the nonlinear process by using nonlinear kernels up to the first order:

(5) $$\begin{array}{c} y\left(t\right)=\gamma_{0}+\overset{d}{\underset{i=1}{\sum }}\gamma_{i}\cdot y\left(t-i\right). \end{array}$$

Motivation is one of the influencing factors, and the nonlinear kernel can be used to measure its influence in the formula. *ϑ*(Mot(*t*)) represents the effect of motivation on emotion transition. The effect of motivation uses Gaussian kernel function equations applied to produce the value corresponding motivation with emotional state:

(6) $$\begin{array}{c} \vartheta \left(\text{Mot}\left(t\right)\right)=\exp\left(-\frac{\text{Mot}\left(t\right)-v_{\text{R}}{\left(t-1\right)}^{2}}{K^{2}}\right), \end{array}$$

where *v*_R_(*t* − 1) is the previous valence value. Thus, the nonlinear kernel can be set as

(7) $$\begin{array}{c} \gamma_{0}=\left(1-\vartheta \left(\text{Mot}\left(t\right)\right)\right)\cdot \text{Mot}\left(t\right),\\ \gamma_{1}=\vartheta \left(\text{Mot}\left(t\right)\right). \end{array}$$

For description of cognitive reappraisal ability, *τ* is used to represent the level of this ability, which is achieved in the sigmoid function:

(8) $$\begin{array}{c} v_{\text{R}}\left(t\right)={\text{sigmoid}}_{\tau }\left(y\left(t\right)\right). \end{array}$$

The sigmoid function can be described as

(9) $$\begin{array}{c} \text{sigmoid}\left(\tau \right)=\frac{1}{1+\left(1-\tau \right)e^{-\lambda x}}. \end{array}$$

### 4.4. Behaviour Layer

Because the emotion regulation influences the sensing-related and the action-related (e.g., behaviour decision) processes, the module contains unconditional response, facial emotional expression based on emotion, and the behaviour based on the collaboration. On the other hand, the input is driven by motivation extraction, emotion generation, or sensory level. The three paths deliver the signal to behaviour decision in parallel. Besides, for excitation from emotion, valence_R_ (in different levels of representations) can trigger emotional facial expressions through stored mapping relationships.

**Figure 6:**
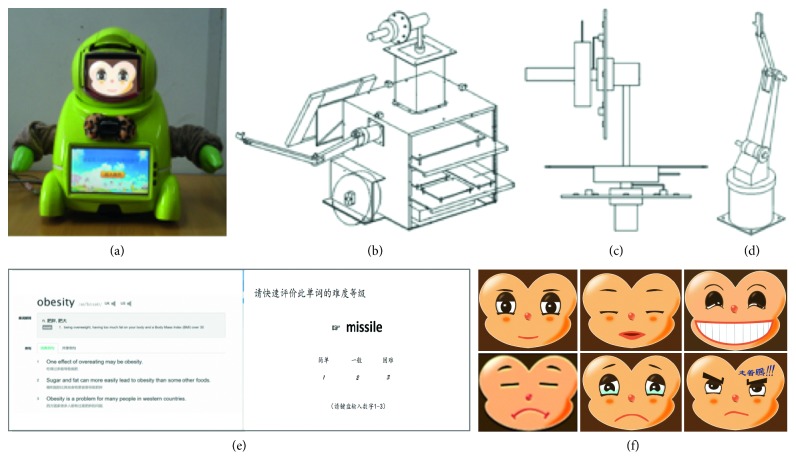
The robotic structure and behaviour: (a) emotional robot; (b) robot's mechanical structure; (c) structure of the neck; (d) structure of the arm; (e) interactive interface for teaching words; (f) emotional expression.

Figure 6 shows the robotic structure and behaviour. The emotional robot is developed by our research group, with 10 DOF. The difficulty level of questions contains three degrees: simple, medium, and hard. More difficult questions answered correctly cause high scores (ranges in 0.6, 0.8, and 1), while more simple questions answered incorrectly cause harsh scores (ranges in 0, 0.2, and 0.4). For behaviours from motivation, student's positive emotion and higher scores can trigger the harder challenge and vice versa. And, for behaviours from robotic emotion, positive teachers' emotion leads to more tolerant teaching methods.

The facial emotional expression ranges in six states (from positive to negative) [45]. In this paper, we did not relate emotional intelligence to expression and provide the corresponding expression output from the internal emotional state for explicit observation instead.

### 4.5. Reinforcement Learning

We use the reinforcement learning [46] method that strives to achieve broad competence in an interactive environment by incorporating internal reward to decide the hierarchical level of agent's emotional influence on behaviour.

Reinforcement learning enables a robot to autonomously discover behaviour outputs under the influence of emotion that chooses difficulty levels of the next question through accumulated reward from the first derivative of emotion. Instead of explicitly detailing the solution to a problem in reinforcement learning, the design of reward provides the competition between motivation and emotion in terms of real-time emotional transition.

Based on the literature presented in the previous studies, the key ingredients of the reinforcement learning setup are observations, goals, and reward design, which are explained as follows:

1. Observations: the robot generates the emotional state after the series processes of cognition-emotion collaboration. Observations at each moment serve not only as the current final emotional state of the robot but also as the source of assessment for reinforcement learning.
2. Goals: the paper focuses on accumulated emotional changes as the outlet of emotional behaviours. Stable changes in robotic emotion prefer to let initial motivation control behaviours. However, when the fluctuation of the emotion is intense, the robot prefers to take “irrational” behaviours controlled by emotional influence.
3. Reward design: it is important to select the time when emotion state is significant enough to control the behaviour decision. We provide the first derivative of emotion as the reward which is a continuous value to represent how much the emotion intensity accumulates:

(10) $$\begin{array}{c} R\left(t\right)=\frac{v_{\text{R}}\left(t\right)-v_{\text{R}}\left(t-1\right)}{\Delta t}\cdot \overset{I_{t}}{\underset{i}{\sum }}\max\left(R\left(t-i\right),0\right), \end{array}$$

where *I*_*t*_ means the final *i* value at the first time max(*R*(*t* − *i*), 0)=0. Winner-Take-All is used to make competitions between the initial motivation and a certain percentage of the reward.

## 5. Results

There are four configurations, compared pairwise, considering two experiment goals: verification of the impact of different robotic preferences and cognitive reappraisal ability on behaviour or its own emotion. C1, C2, C3, and C4 mean configuration 1st (set high preference to emotion), configuration 2nd (set high preference to students' scores), configuration 3rd (set cognitive reappraisal ability to 0.8), and configuration 4th (set cognitive reappraisal ability to −0.8). For obtaining the clear results of the comparison, the first two set the same cognitive reappraisal ability (value = 0), and in the rest two, fair competition is provided between the emotion and scores.

To compare the full system described, a total of 24 interaction processes are performed, 6 for each configuration. In this section, the full system performance is described in the first part, about the robotic motivational and emotional effect on final behaviour decision. Second, we show the impact of different robotic preferences on behaviour or its own emotion. Finally, the effectiveness of cognitive reappraisal is provided. It is noteworthy that we use word “motivation” in the name of initial quantitative motivation, contrasting with emotion, although emotion generation is related to motivation.

### 5.1. Holistic Analysis

Table 1 shows the influence of robotic emotion and motivation on its final behaviour in all 24 experiments. Pearson's correlation coefficient is used for measuring the degree of these influences, which is defined by

(11) $$\begin{array}{c} \rho_{X,Y}=\frac{\mathrm{cov}\left(X,Y\right)}{\sigma_{X}\sigma_{Y}}=\frac{E\left[\left(X-\mu_{X}\right)\left(Y-\mu_{Y}\right)\right]}{\sigma_{X}\sigma_{Y}}, \end{array}$$

where *X* is the robotic reward or motivation vector while *Y* is the selected difficult level of questions representing the behaviour output or the opposite one. Based on correlation coefficient, the correlation between emotion and behaviour is generally lower than the one between motivation and behaviour. This indicates that the behaviour is mainly driven by motivation and we cannot confirm that the emotion did not play a role. As for the influence from motivation to emotion generation, we can see in Table 1 that the correlation coefficients are greater than 0.4, which means motivation has a certain excited effect on emotions but does not hold all influences.

**Figure 7:**
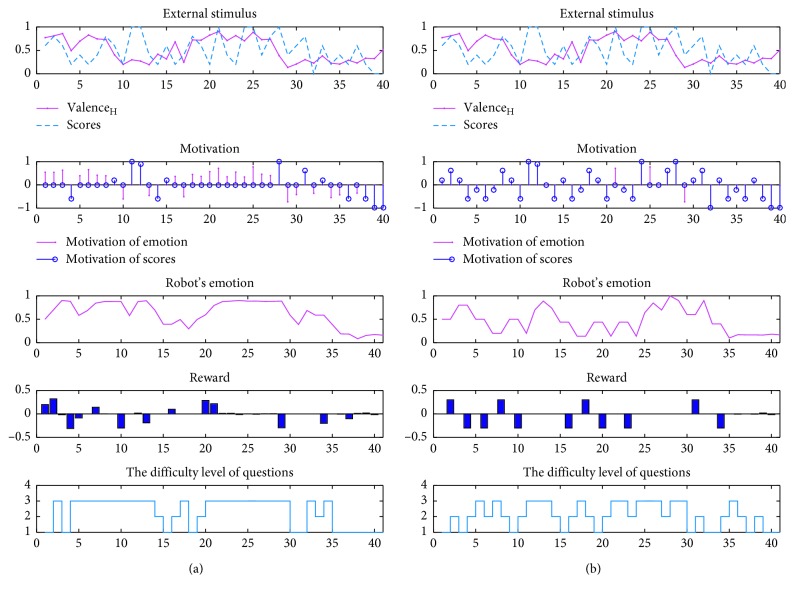
The results during interaction for teaching words task: (a) (C1) preference is set to students' emotion; (b) (C2) preference is set to students' scores.

**Figure 8:**
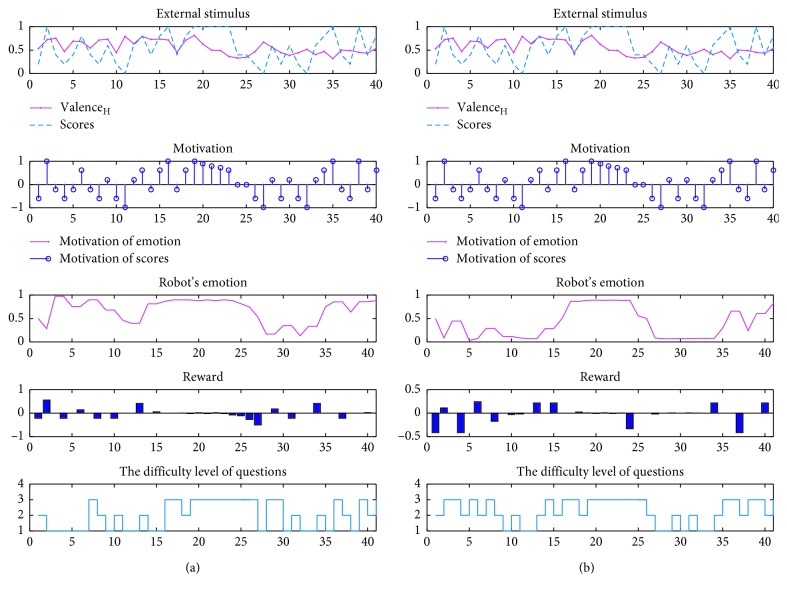
The results during interaction for teaching words task; (a) (C3) cognitive reappraisal ability is set to 0.8; (b) (C4) cognitive reappraisal ability is set to −0.8.

For detailed observation, we provide two typical systematic activity plots: C1 vs C2 in Figure 7 and C3 vs C4 in Figure 8. Take the case of C1, for example:

- For the incipient process, most of the previous external stimuli tend to be positive. Thus, when the agent receives the negative stimulus, it generates a more negative emotional state. We note that the initial decision of the questions' difficulty level has more positive correlation with the agent's emotional state. When motivation continues to decline, the emotional state tends to be more negative. And, motivational fluctuation within a certain range cannot influence the robotic emotional state significantly. It proves the emotion stability during a short time. Moreover, accumulated emotional decline causes the high reward.
- For the middle process, agent's emotional fluctuation tends to be mild although interactive stimulus rises and falls frequently (positive generally). It is noteworthy that the agent prefers to choose more difficult questions once receiving negative scores during the positive condition of students. And, the agent pays more attention to the fluctuation of the student's emotion. During this period, the robotic emotional state has a mild curve, though the external stimulus is being changed. It is worth noting that at the 20th time, the positive stimulus cannot have a significant influence on behaviour, for the reason that robotic emotion wins the competition while robotic positive emotion causes the tolerant decision.
- The later stage of the experiment illustrates that the situation with the high fluctuation of scores and mild human emotional state curve causes motivation about expectation. We can see that continuous negative stimulus causes negative emotional state and negative or weak motivation.

### 5.2. Preference

For intuitive comparison, C1 and C3 provide the systematic activity plot using the data during the real interactive process, while C2 and C4 show the simulation results using the same data recorded by C1 and C3, respectively.

**Figure 9:**
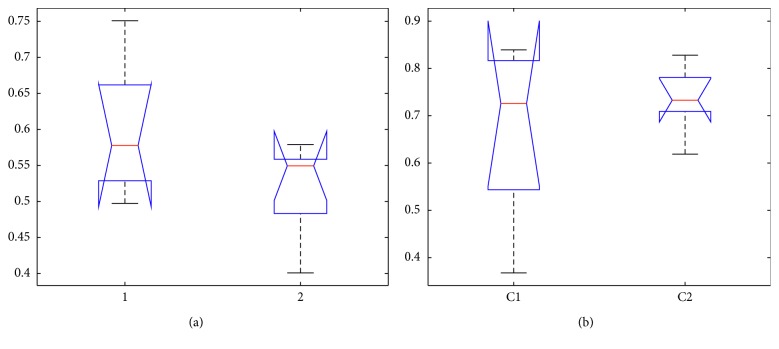
The box plot of configuration 1 vs configuration 2 in which the robot gives preference to (a) students' emotion and (b) scores.

Figure 7 shows the configurations that the robotic teacher pays more attention to scores or student's emotion leading to different emotional state and behaviour decision. Competition ratios are 7 (emotion) : 3 (scores) and 3 (emotion) : 7 (scores). For statistical analysis, Figure 9 shows the nonparametric Kruskal–Wallis (K-W) test between the correlation coefficients of two measures: students' emotion with behaviours (chi square = 0.1, *p*=0.748) and scores with behaviours (chi square = 1.26, *p*=0.2623). And, the Mann–Whitney test has the following results: *U* = 20, *p*=0.409 and *U* = 11, *p*=0.1548. K-W and M-W tests show no significant effect on these configurations, which means the correlation coefficients in C1 or C2 follow the same distribution.

The fact we considered the influence on behaviour regardless of which preference is set does not allow us to conclude on the results, success or failure. Because whatever is students' emotion or score, will cause the motivation, and the K-W and M-W tests can prove that the motivational influence on behaviour follows the definite distribution. And, the effectiveness of these parts can be confirmed by the region of correlation coefficients distribution. In Figure 9(a), the mean of C1 (0.575) is bigger than that of C2 (0.55), and its overall distribution is also higher than that of C2 that confirmed the higher correlation between emotion and behaviours in C1 configuration.

The same reason can be proved in Figure 9(b), in which C1 has 0.72 mean and C2 has 0.73 mean. Though there are similar means, it is obvious that C2 is more concentrated in the high region.

### 5.3. Cognitive Reappraisal

As Figure 8 shows, with the same stimulus and motivation provided, C3 has more positive emotional states than C4. Besides, the selection of difficulty between both is not the same, which proves that the emotion is involved in the decision-making of behavioural output. For verifying the difference between the high cognitive appraisal ability and the low one, the measure is defined as the proportion of first derivative of emotion (>0) to positive motivation:

(12) $$\begin{array}{c} \overset{\to }{\zeta }=\frac{\max\left(v_{\text{R}}^{\left(1\right)},0\right)}{\max\left(\text{Mot},0\right)}. \end{array}$$

**Figure 10:**
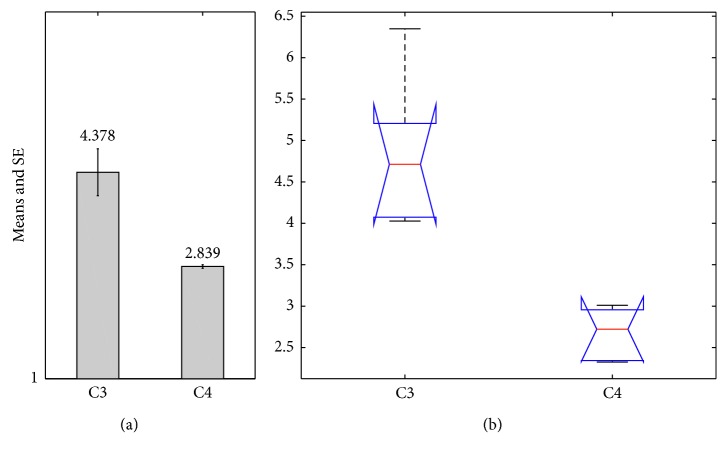
The results of configuration 3 vs configuration 4: (a) mean and SE; (b) box plot.

And, in Figure 10(a), the C1 (mean = 4.57, standard error = 4.378) has higher proportion. The K-W test in Figure 10(b) shows significant effect between the high cognitive appraisal ability and the low one (chi square = 8.31, *p*=0.039). Besides, the M-W test provides the following result: *U* = 0, *p*=0.01, which also proves the effectiveness.

## 6. Discussion

The results presented above highlight the interest of using the effect of emotion-cognition collaboration for a teaching words task. The prototypical behaviours we observe mainly describe four kinds of situations.

### 6.1. For Learners

1. Failure: failure of the learners let the robot generate negative emotion and may provide to select easier question: long-time failure leads to stable negative situations and temporary one just leads to the immediate impact
2. Success: the success of the learners leads to a positive emotion of robots, and the robot may choose more difficult questions for its expectation or may choose more easy questions within pleasant emotional state

### 6.2. For Robots

1. Preference: the robotic teacher has different behaviour preferences when it prefers to care student's emotion or their scores. It proves that the motivation has obvious influence on emotion and behavioural outputs.
2. Cognitive reappraisal: high cognitive reappraisal ability leads to more positive emotion of the robot. The comparison chart shows the emotional influence on behavioural output.

According to the above method, the RECS focuses not only on learners' response score but also on their current state of physiology. Long-term failure will give learners more negative emotions and physical condition. None of these emotional experiences is considered positively. Therefore, emotional regulation is used to avoid these deadlocks and tries to keep the robot in a state of positive development of the emotional state, which could output more positive emotions to students. The evident “instability” of this system is due to interactive stimulus from the variety of uncontrolled circumstance. The impact of robotic emotional influence on behaviours is not obvious because some stable external stimulus conditions have been provided, and the lack of these situations in which bigger enough reward causes the emotional behaviour is also the reason for this. Statistical analysis validates the effectiveness of whatever the preference configuration or cognitive reappraisal configuration. The system provides not only the generation of motivation and emotion but also the emotion-cognition collaboration in terms of behaviour output and emotion regulation.

For teaching tasks, the introduction of emotion regulation strategy is not disjointed with the whole content. Because for intelligentized, humanized robotic teachers, emotion regulation strategy can make the robot more intelligent in the generation of the state of emotion, which considers not only the more positive emotional generation but also the natural emotional transition. Of course, avoiding aforementioned deadlocks also is the major reason.

Though all selections of behaviour from the emotion-cognition influence are the preconditions we set, this paper emphasizes the changeable behaviours and robotic emotion according to the emotion-cognition collaboration. In detailed teaching environments, whether the teacher should avoid certain emotions, attempting to express others, does not allow us to confirm that we should promote positive emotions to exclude negative emotions, or at least not under all circumstances. The complex cognitive processes should need more environment knowledge and more complicated cognitive system. This can be our future research efforts.

## 7. Conclusions

This paper addresses the emotion-cognition collaboration for the teaching words task and focuses on the competition between the motivation and the emotion. As for motivation, the extraction method is provided, and a different robotic preference (personalized part) is considered. As for emotion, we suggest autoregressive time series as the emotional transition framework and introduce the cognitive reappraisal to provide as much positive teaching interaction as possible. The experimental results show the effectiveness of the RECS.

To summarize, the major ideas advocated in this paper are as follows:

- Initial motivational effects consider not only current sensorimotor experience but also the memory. The competition exists not only between different stimulus but also between current and memory.
- Initial motivation can be the stimulus of emotional generation, and emotion transition is also related to the emotion of the previous time.
- Accumulated emotion effects are represented in the rewards in RL as the bargaining counters that compete with the initial motivation.
- High cognitive reappraisal ability can make the robot generate more positive emotion. And, it is meaningful in teaching environments.

## Figures and Tables

**Table 1:** The correlation coefficients of A1, A2, and A3 (A1: motivation and emotion; A2: motivation and behaviour; A3: emotion and behaviour).

Group	Subject	A1	A2	A3
C1	1	0.72	0.80	−0.23
	2	0.78	0.78	0.14
	3	0.72	0.72	0.07
	4	0.63	0.59	0.20
	5	0.74	0.59	0.39
	6	0.74	0.58	0.24

C2	7	0.74	0.78	−0.16
	8	0.74	0.59	−0.05
	9	0.71	0.74	−0.07
	10	0.72	0.62	0.22
	11	0.75	0.83	−0.18
	12	0.70	0.74	0.08

C3	13	0.73	0.83	−0.08
	14	0.60	0.68	−0.07
	15	0.64	0.60	0.13
	16	0.55	0.70	−0.01
	17	0.56	0.58	0.07
	18	0.63	0.47	0.16

C4	19	0.58	0.52	0.14
	20	0.77	0.60	0.15
	21	0.75	0.83	0.04
	22	0.65	0.68	0.17
	23	0.68	0.54	0.10
	24	0.75	0.68	0.16

## Data Availability

The data used to support the findings of this study are available from the corresponding author upon request.
